# Tailoring Formulations for Intranasal Nose-to-Brain Delivery: A Review on Architecture, Physico-Chemical Characteristics and Mucociliary Clearance of the Nasal Olfactory Mucosa

**DOI:** 10.3390/pharmaceutics10030116

**Published:** 2018-08-03

**Authors:** Stella Gänger, Katharina Schindowski

**Affiliations:** 1 Institute of Applied Biotechnology, University of Applied Sciences Biberach, Hubertus-Liebrecht-Strasse 35, 88400 Biberach, Germany; stella.gaenger@gmx.de; 2 Faculty of Medicine, University of Ulm, Albert-Einstein-Allee 11, 89081 Ulm, Germany

**Keywords:** olfactory epithelium, respiratory epithelium, NALT, semisolid, nanoparticles, CNS drug delivery, biopharmaceuticals, dosage form, medical device

## Abstract

The blood-brain barrier and the blood-cerebrospinal fluid barrier are major obstacles in central nervous system (CNS) drug delivery, since they block most molecules from entering the brain. Alternative drug delivery routes like intraparenchymal or intrathecal are invasive methods with a remaining risk of infections. In contrast, nose-to-brain delivery is a minimally invasive drug administration pathway, which bypasses the blood-brain barrier as the drug is directed from the nasal cavity to the brain. In particular, the skull base located at the roof of the nasal cavity is in close vicinity to the CNS. This area is covered with olfactory mucosa. To design and tailor suitable formulations for nose-to-brain drug delivery, the architecture, structure and physico-chemical characteristics of the mucosa are important criteria. Hence, here we review the state-of-the-art knowledge about the characteristics of the nasal and, in particular, the olfactory mucosa needed for a rational design of intranasal formulations and dosage forms. Also, the information is suitable for the development of systemic or local intranasal drug delivery as well as for intranasal vaccinations.

## 1. CNS Drug Delivery

### 1.1. Chalenges in CNS Drug Delivery

Drug delivery technologies such as liquid, semi-solid or particulate formulations are important to deliver pharmaceutical compounds safely to their desired site of therapeutic activity [1]. A highly critical point in drug delivery is the low availability of drugs in the central nervous system (CNS) due to the blood-brain barrier. This barrier prevents 95% of molecules from entering the CNS by numerous tight junctions and efflux transporters [2]. Though, the blood-brain barrier is more permeable for small lipophilic molecules and provides some specific transporters, it limits severely therapies for CNS diseases [3,4].

The current state-of-the-art to deliver drugs with a low central bioavailability is intrathecal, intracerebroventricular or intraparenchymal injections that deliver directly to the cerebrospinal fluid (CSF) of the CNS. A chronic drug supply can be provided by implanted medical devices. Although, such techniques are in clinical use, these routes of administration are invasive and their use is predominantly limited to intensive care as the risk of infections is not neglectable [5,6,7,8,9]. Hence, a safe and efficient drug delivery platform technology for CNS active molecules is needed. 

### 1.2. Intranasal CNS Delivery and Some Clinical Evidences of Its Applicability

**Figure 1:**
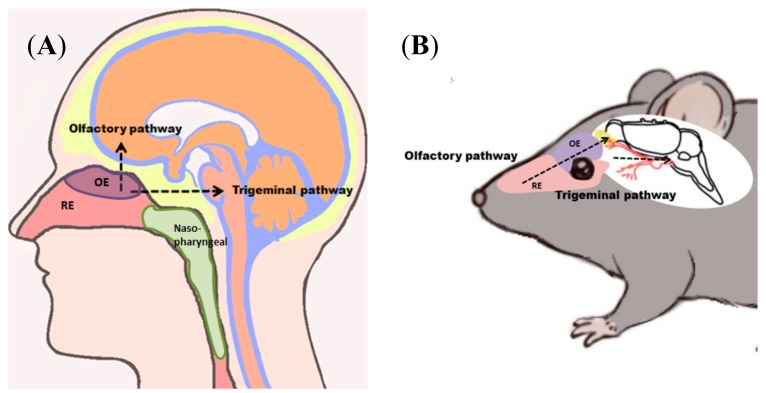
Nose-to-brain (N2B) delivery allows application of drugs at the roof of the nasal cavity, which are transported to the central nervous system (CNS) of humans (**A**) and rodents (**B**). N2B transport can either be mediated via the olfactory or the trigeminal pathway. Drugs passing along the olfactory pathway target the olfactory bulb, whereas drugs transported via the trigeminal pathway are delivered predominantly to the brain stem. Abbreviations: OE: olfactory epithelium/mucosa; RE: respiratory epithelium/mucosa.

In general, the nasal cavity is highly suitable for drug delivery as the nasal mucosa presents an efficient absorption and a very good permeability of both: small molecule drugs and biopharmaceuticals. Furthermore, the roof of the nasal cavity is located in very close vicinity to the brain (skull base) and harbours nerves that project to the brain. Therefore, a promising strategy to bypass the blood-brain barrier and blood cerebrospinal-fluid barrier is the delivery of drugs from the nose to the brain (N2B; Figure 1). N2B delivery is minimally invasive with a decent patient compliance and the potential for self-medication. The N2B drug route is discussed to deliver substances to the brain via the olfactory and trigeminal nerve. N2B is not limited to small molecule drugs, as peptides or proteins, even stem cells, viruses and nucleotides have already been proven to pass from the nose to the brain. [3,10,11]. 

N2B delivery of substances would provide a direct and minimally invasive way of targeting the site of action in neurological diseases like Alzheimer’s or Parkinson’s disease [4,12], mental disorders or other diseases like cancer or viral infections. The best-known drugs that have been investigated for intranasal drug delivery is probably oxytocin and insulin [13,14]. Intranasally applicated insulin can decrease hyperglycemia in type I diabetes, improves the outcome of healthy volunteers in some cognitive task and is investigated as cognitive enhancer in Alzheimer’s disease [13]. According to the U. S. National Library of Medicine (ClinicalTrials.gov) there are more than 30 clinical studies with intranasal insulin had been performed, are currently ongoing or are planned. (15.07.2018 https://clinicaltrials.gov/ct2/results?term=Nasal+Insulin&Search=Search).

### 1.3. Importance of CNS Drug Delivery

The therapy of CNS diseases like neurodegenerative, demyelinating or psychiatric diseases as well as brain tumors is still an unmet medical need due to the low CNS bioavailability of innovative drugs like antibodies. One of the diseases with highest mortality rate still is cancer. A major problem in brain tumor treatment is drug targeting via the blood-brain barrier to the tumor site. Therefore, invasive intracerebroventricular or intraparenchymal injections are used. A currently investigated option is the use of targeting receptors such as transferrin to induce transcytosis in the blood-brain barrier: The targeting ligands can be either fused to biopharmaceuticals or are on the surface of nanoparticles [15]. However, the adverse effects of using transferrin for targeting need to be studied as the endogenous transferrin metabolism could be affected. As intranasal administration bypasses the blood-brain barrier, targeting strategies could be enhanced to cell-specific targeting. Ligated nanoparticles specifically targeting the tumor site would be of great interest, as the drug still needs to be directed to the tumor. The combination of dual targeting delivery systems with intranasal application would maximize the chance of drugs reaching the site of action.

Several viruses are able to infect the CNS, but anti-viral drugs are on the other hand efficiently hindered in penetrating the blood-brain barrier by efflux transporters. N2B delivery may provide a new opportunity for the delivery of anti-viral drugs to their site of action. Amongst others it would especially be useful for the treatment of human immunodeficiency virus (HIV), as CNS macrophages are one of the most important HIV hosts in the human body [16].

## 2. Drug Delivery via the Nasal Cavity

### 2.1. Anatomy of the Nasal Cavity

**Figure 2:**
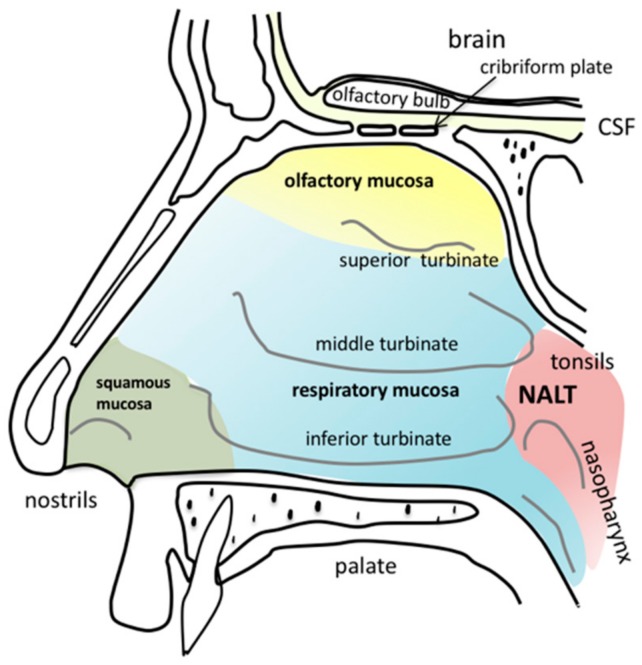
Anatomy of the human nasal cavity. Squamous mucosa (green) is located at the frontal parts of the nasal vestibules. The three turbinates (inferior, middle and superior) humidify and warm the inhaled air. The area covered predominantly with respiratory mucosa is labelled in blue. The olfactory mucosa (yellow) is located next to the cribriform plate at the skull base down to the superior turbinate. Nasally transmitted substances can cross the cribriform plate via different pathways to enter the brain. Nasopharynx-associated lymphatic tissue (NALT) is located in close proximity to the tonsils at the nasopharynx.

The human nasal cavity extends from the nostrils to the nasopharynx corresponding 12–14 cm in length and is divided by the nasal septum. Three turbinates, the inferior, the middle and the superior turbinate, with an overall mucosal surface area of about 160 cm^2^ filter, warm and humidify the inhaled air [3,17]. The nasal cavity harbours four different types of epithelia and underneath mucosa: squamous, respiratory, transitional and olfactory (Figure 2). Squamous mucosa can be found at the frontal parts of the nasal vestibule. The nasal vestibule extends from the nostrils to the inferior turbinate. Squamous epithelium is stratified and its mucosa contains hairs, sebaceous and sweat glands. Transitional epithelium has no cilia and is the one separating the squamous from the respiratory epithelium and the respiratory from the olfactory epithelium, respectively. Regarding drug absorption, the respiratory and olfactory mucosa are of particular interest [3,17,18]. Hence, they will be described in detail below. The nasopharynx-associated lymphoid tissue (NALT) contains immune cells and is in humans highly connected to local lymph nodes and tonsils.

### 2.2. General Suitability of the Nasal Cavity for Drug Delivery

Most types of nasal mucosa are involved in drug absorption, but interestingly, they differ in their delivery routes and their targeting compartment (Table 1). 

Until now, there are hardly any medical devices on the market that are able to specifically target only one delivery pathway. In addition, tailored mucoadhesive formulations have a huge potential to specifically target the desired type of nasal mucosa thereby enabling preferentially one drug route. To develop appropriate intranasal drug formulations, the physico-chemical characteristics, the cytoarchitecture and the mucociliary clearance of the nasal mucosa are important criteria. Therefore, we review here first the composition of the nasal olfactory region, then we take a close look at N2B pathways and their implications for intranasal drug formulations.

## 3. The Nasal Epithelia

### 3.1. Olfactory Mucosa

**Figure 3:**
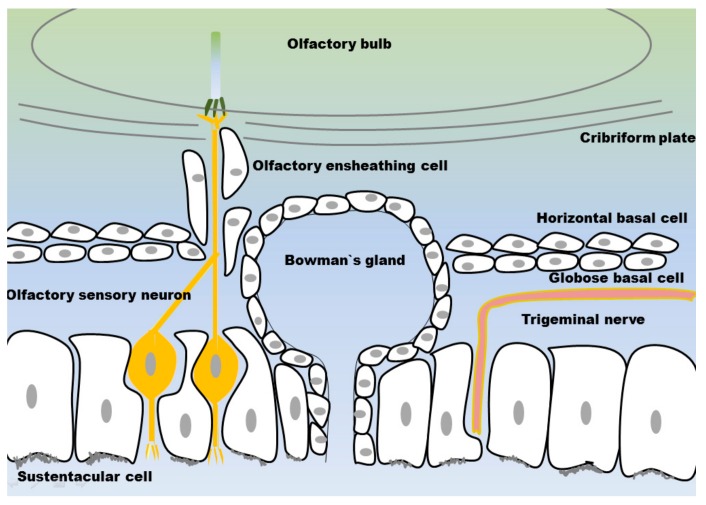
Structure of the olfactory mucosa: the olfactory sensory neurons (OSN) and the trigeminal nerve are embedded in a layer of supporting cells (sustentacular cells) and Bowman’s glands. Horizontal and globose basal stem cells are embedded in the lamina propria. The OSN axons are surrounded by olfactory ensheathing cells and form neuronal bundles. The neuronal bundles penetrate the cribriform plate and extend into the olfactory bulb.

The mucosa covering the olfactory region is composed of the neuronal cells detecting odorants in the inhaled air. The neurons are surrounded by either supportive cells in the epithelial layer and cells ensheathing the olfactory axons in the lamina propria on their way to the olfactory bulb. Basal stem cells ensure the recovery of olfactory mucosa after injury or tissue maintenance related cell death. Bowman’s glands produce and secrete mucus. The various cell types and structures are sketched in Figure 3 and will be described in detail below. 

#### 3.1.1. Olfactory Sensory Neurons

The olfactory nerve is the so-called first cranial nerve. The olfactory system is unique as the olfactory nerve shows some atypical features. Olfactory sensory neurons (OSN) are non-myelinated neurons enwrapped by specialized ensheathing cells. Axons projecting from the olfactory nerve do not from a single bundle, as other nerves do. OSN form glomeruli, the so-called fila olfactoria, which project in bundles to the olfactory bulb [26]. OSN are exposed to the inhaled air and thereby in direct contact to airborne noxious substances as germs, pollutants or toxic agents. Therefore, it is assumed that the short lifespan of about one month and systematic apoptosis of OSN are mechanisms to protect the brain from infections. For maintenance, the olfactory mucosa contains two different types of basal stem cells [27,28] differentiating to OSN [26]. The dying OSN leave gaps in the epithelial layer until a new OSN regrows into the same space. During their replacement there is a delay in tight junction formation [11]. 

Non-motile primary cilia cover the OSN are on their apical side and harbour the olfactory receptors. Binding of exogenous odour molecules is transduced into electrochemical stimulation, which travels along the olfactory axons to the brain [29,30]. The mammalian olfactory system is able to detect thousands of odorants. The OSN express olfactory receptors, which recognize specific odours. Humans express up to 400 different olfactory receptors, while there are even up to 1200 different receptors in rodents [31]. The one-neuron-one-receptor rule indicates that one OSN is exclusively expressing one olfactory receptor [31,32]. The axons of OSN which express the same olfactory receptor converge to form neuronal bundles projecting to the olfactory bulb. There are various glomeruli, each of them projecting to an array of 10–20 mitral/tufted cells located in the olfactory bulb and projecting to higher brain regions [33,34]. 

#### 3.1.2. Olfactory Ensheating Cells

Olfactory ensheathing cells (OEC) surround and isolate the olfactory axons from their origin in the epithelial membrane up to the lamina propria, where these axons form glomeruli with axons from matching OSN. In the outer layer of the olfactory bulb, olfactory ensheathing cells enwrap axonal bundles from OSN, where they defasciculate and finally terminate into olfactory bulb glomeruli [35]. 

The diverse expression patterns of OEC at different localizations imply the presence of different types of OEC: OEC resident in the lamina propria (LP-OEC), OEC present along the outer olfactory nerve fibre layer (NFL-OEC) and OEC located in the inner olfactory nerve layer on the olfactory bulb (OB-OEC). OEC located in the mucosa mainly interact with each other by adhesion. They guide several axons to form bundles, whereas the OEC of the olfactory bulb do have a broader spectrum of interaction. The OB-OEC may interact by adhesion or by repulsion or simply do not interact at all. OB-OEC also do not cause fasciculation of axon bundles [35,36,37]. 

OEC do not only maintain the electrophysiology of mature OSN, they also contribute to a great extent to regeneration. They provide the environment needed for neurite outgrowth and assist in the setup of new functional synapses. In contrast to that they take also part in the controlled death of OSN [35,36]. Additionally, OEC are currently discussed for their potential use in regeneration of spinal cord lesions. Although OEC do not normally myelinate OSN, they are able to remyelinate damaged axons to restore physiological function [35,36]. Chuah et al. showed that if the olfactory mucosa is damaged, OEC are contributing to host defence by nitric oxide production, leading to an increase of inducible nitric oxide synthase (iNOS). Furthermore, chemokines like interleukin-6 (IL-6), monocyte chemoattractant protein 1 (MCP-1), chemokine C-X-C motif ligand 1 (CXCL1) and tumour necrosis factor-alpha (TNF-alpha) were elevated to initiate an immune response. This could not prevent pathogen invasion in total, but decreased its extent and prevented spreading to the deeper levels of the olfactory bulb. Also, OEC and supporting cells produced pituitary adenylate cyclase-activating peptide (PACAP), a protein protecting against TNF-alpha induced cell death by activating anti-apoptotic pathways [38,39]. Based on this and other findings it is hypothesized that microglia of the olfactory bulb have a low activation threshold, lower than that of other brain regions. The immunological function of OEC and of olfactory bulb glial cells need to be considered when developing intranasal N2B biopharmaceuticals. If the drug enters the nervous system via the olfactory pathway there may be an activation of olfactory bulb microglia, which may lead to an inflammatory response throughout the brain. OEC induced immune response may also have an effect on the distribution and internalization of the drug [38,40].

#### 3.1.3. Sustentacular Cells

Sustentacular (SUS) cells belong to the group of so-called supportive cells. SUS cells show characteristics of both, epithelial and neuronal cells. Their particular role and function still needs to be elucidated. While OEC wrap around OSN axons, SUS are enclosed around OSN dendrites, to ensure structural stability and to maintain ionic integrity [41,42]. Enwrapping occurs with OSN maturation: while most of the immature OSN dendrites are unwrapped, the majority of mature OSN dendrites are enwrapped with SUS [41]. Juxtanodin, an actin cytoskeleton-related protein, is present in SUS that plays also an important role in forming the myelin sheaths of oligodendroglia in the CNS. It is thought that juxtanodin regulates the interaction of SUS cells with OSN in the same way as it is described for oligodendroglia. Cell membranes of SUS enwrapping adjacent OSN contain gap junctions [41,43]. SUS do not only enwrap mature OSN dendrites, they also provide an environment for regeneration. Immature OSN migrate alongside SUS during maturation [41]. 

Endothelin signaling provides a way of SUS and OEC damage control [42]. Endothelin also acts as survival factor, it uncouples gap junctions between SUS enwrapping adjacent OSN to limit cell death [42]. 

#### 3.1.4. Globose Basal Cells

Globose basal cells (GOB) are active cycling stem cells frequently differentiating into neuronal as well as non-neuronal cells for the regeneration of the olfactory mucosa. GOB maintain renewal of the mucosa in normal tissue homeostasis and also in response to injury. Proliferation of GOB cells is stimulated by dying neurons releasing leukemia inhibitory factor (LIF), or nitric oxide release during inflammation or cell death. Fibroblast growth factor-2 (FGF-2) also stimulates GOB cell proliferation. Proliferation of stem cells is regulated through local cell density via a negative feedback [28,44,45].

#### 3.1.5. Horizontal Basal Cells

Horizontal basal cells (HBC) are mainly quiescent basal stem cells. They only take part in olfactory mucosa maintenance in case of extensive tissue injury. It is supposed that GOB differentiation is sufficient for mucosa preservation and HBC are only needed in case of lager injuries. HBC and GOB are both able to replace neuronal as well as non-neuronal cells. Behringer et al. suggest a more prominent role for HBC in maintenance: HBC show waves of activity soon after birth as well as after tissue injury. The authors also assume that HBC give rise to new GOB and if needed take also part in cell replacement by differentiating into OSN, Bowman’s glands or SUS [45]. In contrast to GOB, HBC have primary cilia, which may play a role in communication with SUS [44]. Proliferation of HBC is stimulated by epidermal growth factor (EGF) and transforming growth factor alpha (TGF-alpha) [28].

#### 3.1.6. Bowman’s Glands

Bowman’s glands are located in the lamina propria of the olfactory mucosa. They have a simple tubular structure with no branches. Vesicles are located at the apical pole, close to the lumen. The nucleus is located at the basal pole where a few microvilli are present. Two different kinds of secretory vesicles were observed in Bowman’s glands. One type appears to contain mucus glycoprotein as MUC5AC and the other vesicles are packed with proteinaceous and serous content [46,47,48,49]. Aquaporins (AQP) AQP1, AQP3, AQP4 and AQP5 pump water needed for the olfactory mucus. The ducts of Bowman’s glands transmigrate the OE. In other areas of the nasal cavity, secretory glands do not penetrate the epithelium to the surface, they secrete via duct systems to the nasal vestibule [46,50,51]. Bowman’s glands are surrounded by multiple bundles of OSN axons. Furthermore OEC, fibroblasts and large bundles of collagen fibres are observed in the entourage of Bowman’s glands [46]. 

The exact composition of the olfactory mucus secreted from Bowman’s glands is still unknown [46]. Histological approaches showed, that these glands are positive for periodic acid-Shiff (PAS) staining which indicates neutral glycoproteins. In another study sulphated glycoproteins were found. Acidic glycoproteins were identified with Alcian blue staining [46,52,53]. Septal nasal glands and submucosal glands in the lower respiratory tract express a chloride channel at the apical site of secretory cells (cystic fibrosis transmembrane conductance regulator protein, CFTR), which regulates secretion. This regulatory protein is not present on Bowman’s glands and, thus, the secretion mechanisms are yet unknown [46,54].

### 3.2. Respiratory Mucosa

About 80–90% of the human nasal cavity is covered with respiratory mucosa, which warms the inhaled air and does also take part in first line defence as it filters the air and removes particles such as allergens and microorganisms. The human respiratory mucosa is composed of various cell types and glands. Goblet cells, ciliated cells, intermediate cells, basal cells, serous glands, seromucous glands and intraepithelial glands can be found [3]. Most of the nasal secretions are produced by the seromucous glands. Basal cells serve as progenitor cells in the respiratory mucosa [3]. Like olfactory mucosa, respiratory mucosa is innervated by the trigeminal nerve [3]. Seromucous glands produce mucus, but also secrete antibacterial proteins for immune defence [55]. In addition to that, mucus is also secreted by goblet cells as previously described in the gut [3,56]. Up to now, there is not much known about the glands in the respiratory mucosa. Most information of glands as seromucous glands or goblet cells originate from the upper and lower airways or the gut. Olfactory mucosa lacks goblet cells [46]. Respiratory as well as olfactory mucosa is covered with mucus. As their name already suggests, the task of ciliated cells present in the respiratory mucosa is to propel mucus towards the nasopharynx, where it is swallowed and digested [3]. 

Both, the large surface area and the high vascularity of the respiratory mucosa make it interesting for systemic drug application. It appears that CNS delivery via the respiratory mucosa is limited to the trigeminal nerve pathway [11].

### 3.3. Nasopharynx-Associated Lymphatic Tissue

As all mucosal surfaces are in direct contact with pathogens, the mucosa-associated lymphoid tissue (MALT) is an important part of every mucosa to protect the organism from infections. The different MALTs are interconnected as the induction of an immune response at one mucosa may result in IgA production on another mucosal site in a different organ. MALT is even able to act independently of the systemic immune system [57]. Immune responses of MALT are induced in secondary immune tissues, where antigen sampling is preceded beforehand. Nose specific MALT is called nasopharynx-associated lymphatic tissue (NALT). The site, where immune function is maintained, is composed of diffusively distributed lymphatic tissue along the lamina propria. It contains T-cells, IgA-, IgM- and IgG-secreting plasma cells, few B-cells and dendritic cells as well as macrophages and specialised Microfold cells [57,58]. Specific T cell activation leads to clonal expansion of B cells and the production of secretory IgA, but also IgG [57,59]. NALT resident B cells secrete IgA via T_H_2- dependent interactions. In addition, NALT is also able to mediate immune tolerance through T_H_1 and cytotoxic T cell mediated reactions [57]. The mucosal immune system can be divided into the inductive and the effector sites: the inductive site is the region where antigen sampling occurs and lymphocytes are primed, while the effector site comprises mainly of the epithelial surface and the lamina propria where the immune cells perform their activity [60,61]. 

Besides the NALT, primates obtain at least three sets of tonsils. Since in human airways particles impact primarily at the pharynx, the Waldeyer’s tonsillar ring surrounds the pharynx [59]. Tonsils can be divided in two groups, the one with and the one without crypts. The tonsils with crypts are projecting to the surface of submucosal lymphoid tissue, whereas the ones without emerge into the oro- or nasopharynx [57]. The reticular epithelium overlying the tonsils with its lymphoid follicles contains Microfold cells, lymphocytes, macrophages, dendritic cells and some granulocytes. The non-reticular epithelium separates the reticular epithelium [57]. 

#### Microfold Cells

Microfold cells (M cells) are present in lymphoid follicle-associated epithelium. Differentiation of M cells from their progenitor stem cells is induced by RANKL-RANK signalling. NALT resident M cells have a lifetime of weeks or even up to months, whereas intestinal M cells only survive several days [62,63]. M cells display unique characteristic features which distinguish them from other lymphatic tissue cells like their high endocytotic activity, irregular brush order, reduced microvilli and the absence of cilia on their cell surface, thin glycocalyx, sparse lysosomes and, finally, their typical basolateral pockets [60]. M cells are specialized cells for the phagocytosis and transcytosis of particles and antigens that are present in the mucus. They are also able to transport whole virus, bacteria or even parasites across the epithelium [60,62,64,65], but M cells themselves do not present exogenous antigens [63]. Once engulfed and transported across the follicle-associated epithelium, M cells direct the phagocytosed particles into their intra-epithelial pockets beneath their basolateral membrane. The pockets contains different immune cells like lymphocytes, macrophages and dendritic cells and provide a specialized environment for their interaction [58,64]. Thus, M cells pass their sampled content to dendritic cells, which present the antigens to lymphocytes [64]. It is thought that M cell sampling occurs both, in a selective and non-selective manner. Pappo and Ermak could show that M cell transcytosis is very fast. Fluorescent latex particles were initialized by the M cells within 10 min after administration. Transmembrane transport by M cells is at least 50 times faster than the transport by their adjacent cells [66]. Some bacteria and viruses have developed the ability to specifically use M cell transport to invade the host [64]. M cells are also able to transcytose secretory IgA and IgA immune-complexes [64].

M cells are supporting the efficacy of intranasal vaccinations. Such intranasal vaccinations provide the benefit of systemic immunity without the need of parenteral injections [67]. M cells are able to transcytose the immunogenic agents very fast and pass them to the underlying lymphatic tissue. Here, the immune cells are primed and a specific immunity against the vaccine is induced [60,64]. To facilitate and speed up uptake, M cell receptors may be specifically targeted by fusing the vaccine agent with receptor ligands [60]. When developing intranasal delivery for therapeutic proteins, the interaction of the biopharmaceuticals with M cells in particular and NALT in general needs to be carefully considered to avoid immunogenicity. 

### 3.4. Trigeminal Nerve

OSN are not the only neurons invading the nasal mucosa. The trigeminal nerve is also innervating the olfactory and respiratory mucosa to a certain extent. Thus, chemical stimuli entering the nasal mucosa do not only interact with OSN, but also with trigeminal chemoreceptors. The trigeminal nerve is the fifth and largest of the cranial nerves and innervates the brain stem in the region of the medulla, pons and spinal cord, respectively [68]. It has three branches, the ophthalmic, the maxillary and the mandibular one. The ophthalmic and the maxillary branches innervate the nasal cavity. The nasopalatine nerve, which is innervating the posterior portion of the nasal cavity belongs to the maxillary branch, while the ethmoid nerve innervating the anterior nasal mucosa and external nasal surface belongs to the ophthalmic branch [69]. In contrast to the axonal bundles of OSN, the trigeminal nerve is predominantly myelinated with Schwann cells. Non-myelinated branches of the trigeminal nerve innervate blood vessels of the olfactory mucosa to regulate blood flow [35]. Trigeminal nerve endings are found in the nasal epithelium beneath the line of the tight junctions. Thus, trigeminal nerve endings are not penetrating the surface of the epithelium like OSN. Trigeminal innervation leads to sensory perception such as touch and pain. Solitary chemoreceptor cells are supporting the detection of water soluble irritants. Stimulation of trigeminal nerves by irritants is indicated by the sensation of stinging, burning or pain. It also activates protective reflexes such as increased secretions of nasal mucus or a decrease in size of the nasal passage [69,70,71]. Cell bodies of trigeminal nerves are found on trigeminal ganglions [69]. It was shown by Ekberg et al. that the bacteria *Burkholderia pseudomallei* are able to infect the brain stem directly via the trigeminal nerve within 24 h [72]. The mechanism of bacteria invading and traveling along the trigeminal nerve is also interesting for drug delivery aspects, notably for particulate formulations.

### 3.5. Tight Junctions

Like in all epithelia, tight junctions seal the space between the different apical cells and prevent exogenous molecules from entering the mucosa [73]. Tight junctions are mainly composed by the protein occludin, the protein families claudin and zonula occludens. But not only the apical side is shielded by tight junctions, adherence junctions are also observed on fila olfactoria in the lamina propria. Occludin was found as well on endothelial cells of blood vessels in the lamina propria [74]. In addition, tight and adherence junctions are also present in Bowman’s glands [75]. Tight junctions of the olfactory mucosa do not only prevent foreign particles from entering the CNS, they also provide a milieu for axonal growth thanks to the micro-compartmentalization of fila olfactoria. Tight junctions are able to compartmentalize axonal bundles by adjusted leakiness and thus may take part in an environment for axonal regrowth [73]. This is supporting the theory of adherence junctions playing a role in the maintenance of neuroplastic processes [75]. 

Cell-cell connections like tight and adherence junctions decrease the permeability of drugs through the mucosa. Nevertheless, their presence does not directly reflect the permeability of the mucosa. Despite the presence of tight junctions, the nasal epithelia provide a low transepithelial electrical resistance (TEER) and a good permeability for drugs [2]. Manipulation of tight junctions forming the blood-brain barrier is discussed to facilitate drug delivery into the CNS. But a leaky blood-brain barrier did not only enhance drug delivery, but also the risk of CNS infection and may lead to severe side effects as the increased risk of brain oedema [2]. In parallel, manipulation of tight junctions in the olfactory mucosa may also cause irreversible damage. One of the substances with an apparent limited risk is papaverine, a vasodilator with time dependent reversible effect on tight junctions [2].

## 4. Cilia, Nasal Mucus and Mucociliary Clearance

When developing formulations (e.g., liquid formulations, nano- or microparticles, semi-solid formulations) for intranasal delivery in general and for N2B delivery in particular, mucus composition, clearance and renewal have to be carefully considered [3,68,76]. This will enable to tailor a formulation to the needs of the local application area.

### 4.1. Cilia and Mucus Transport

**Figure 4:**
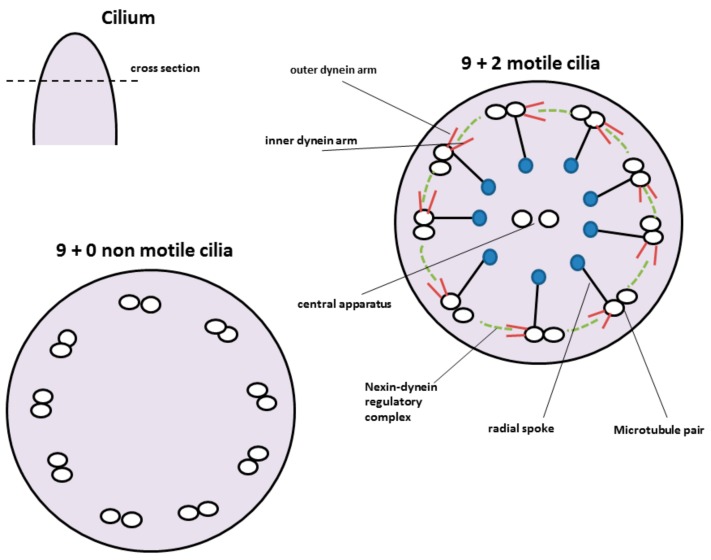
Structure of motile and non-motile cilia. Motile cilia show a cartwheel like structure with nine microtubule pairs surrounding a central pair. The outer microtubule pairs are connected via radial spokes to the central one. Motility of the cilium is provided by the nexin-dynein motor complex. Non-motile cilia lack the central microtubule pair as well as the nexin-dynein motor complex.

There are two types of cilia known, the motile and the non-motile ones. Non-motile as well as motile cilia share a common scaffold. They consist of a skeleton called the axoneme made up of hundreds of proteins. The interior of the axoneme is composed of nine peripheral microtubules. These microtubules are arranged in a cartwheel like formation and consist of doublets, composed of A and B tubules. These nine microtubules either surround a central pair of microtubules (9 + 2) or they lack the inner one (9 + 0); 9 + 2 cilia are motile and occur as multiple cilia, whereas 9 + 0 cilia can be motile or non-motile (Figure 4). Almost every cell has a single, non-motile primary cilium, whereas only specialized cells display multiple cilia [77].

The cilial beat pattern consists of two phases, the effective stroke and the recovery stroke. The effective stroke counters viscose resistance and propels the mucus, respectively. The recovery stroke brings the cilium back to its starting position. To avoid viscous resistance as good as possible the cilium moves in a tangential motion close to cell surface. The motion of motile cilia is dynein-motor based. Energy is provided through ATP hydrolysis. Dynein attachment and activity is regulated by Nexin-dynein regulatory complexes, which link microtubule doublets [77,78]. Dynein motor complex is present in motile cilia only and consists of various proteins.

Nearly 80% of the cells of the respiratory mucosa are covered with motile cilia. In the lower airways over 99% of the cells are ciliated, while in olfactory mucosa only non-motile cilia are found. However, smaller patches and islets of respiratory mucosa can be found in the olfactory mucosa [46,79]. Such respiratory islets contain motile cilia and support the mucociliary clearance of the olfactory region. 

### 4.2. Nasal Mucus and Clearance

#### 4.2.1. Biochemical Structure of the Mucus

The mucus consists predominantly of proteins of the mucin family and water. Mucins share a high homology, but it is still not completely elucidated, if several splice variants or individual genes are expressed [80,81]. Airway mucins are either secreted, oligomeric gel forming mucins as MUC2, MUC5AC, MUC5B, MUC19 or a secreted monomeric non-gel forming mucin like MUC7. The majority of mammalian mucins is membrane bound [80] like the surface associated mucins MUC1, MUC16 and MUC20, which are located on the apical side of epithelial cells [82]. MUC5AC is the most prominent mucin present in healthy airways. When it comes to chronic diseases as cystic fibrosis or chronic obstructive pulmonary disease, the ratio changes and MUC5B is the dominant mucin [3,82,83]. Roy et al. showed, that MUC5B is primarily in charge of airway defence while MUC5AC is less involved [82,84]. From all mucins found in the airways, only MUC5AC is observed in olfactory mucosa [82]. Mucins show putative antimicrobial and immunomodulatory effects and may have evolved differently from each other to maintain host environment-specific immune reactions [80,85]. 

**Figure 5:**
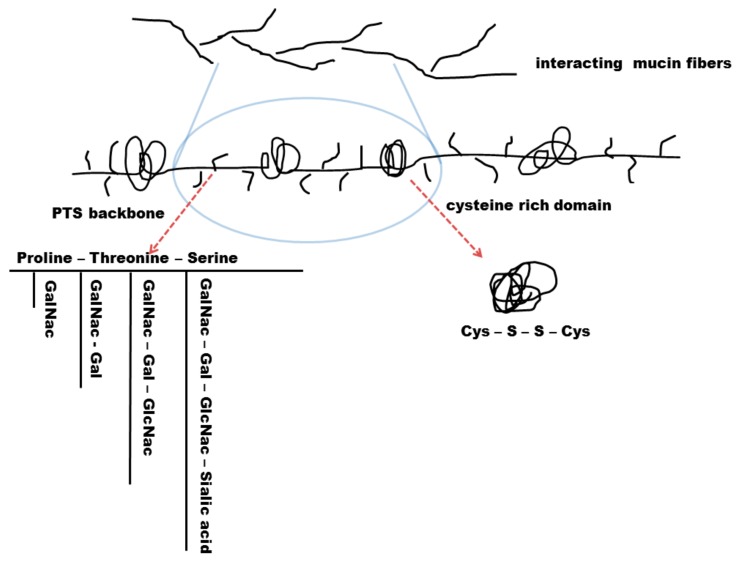
Secreted mucin fibres have a common scaffold consisting of a recurring PTS backbone (proline-threonine-serine) with intermitting cysteine rich domains. These domains are coiled due to their disulphide bond interactions. PTS backbones contain different amino sugar glycosylation such as *N*-Acetylgalactosamine (GalNac), *N*-Acetylgalactosamine-Galactose (GalNac-Gal) glycosylation, *N*-Acetylgalactosamine-Galactose-*N*-Acetylglucosamine (GalNac-Gal-GlcNac) glycosylation, or *N*-Acetylgalactosamine-Galactose-*N*-Acetylglucosamine-Sialic acid (GalNac-Gal-GlcNac-Sa) glycosylation. The degree of glycosylation influences mucus permeability and viscosity.

All mucin fibres display so called PTS (proline, threonine and serine) regions that are highly glycosylated via O-linked bonding (Figure 5). Membrane bound mucins also contain N-linked sulphate groups at the end of each mucin fibre. SEA domains (sea-urchin sperm protein, enterokinase and arginine) are adjacent to the transmembrane binding sites of membrane bound mucins and are able to auto-proteolyse when shear stress occurs. It seems as this is a mechanism of mucin shedding without destroying the cell membrane [86]. 

Gel-forming mucins each have a length of 0.2–0.6 µm and are linked to other mucin fibres via disulphide bonds. Prior to their secretion, mucin granules are packed densely with H^+^ and Ca^2+^ ions. H^+^ ions neutralize excess negatively charged carboxyl groups while Ca^2+^ crosslinks the remaining glycans. When H^+^ and Ca^2+^ diffuse after mucin secretion, mucin fibres entangle to a certain extent and the volume of the mucus gel rises. This process takes only about 50 ms and leads to a 500-fold increase of the mucosal volume [87,88]. The very hydrophobic region of secreted mucins consists of 110 amino acids with a region of ten cysteines in a row. The cysteine region forms hydrophobic coils and it is thought to be the equivalent to the SEA domain in membrane bound mucins. Secreted mucins display several of those hydrophobic regions which are separated by recurring PTS domains [86,89,90]. The negative charge of glycans ensures hydrophilic properties of the mucins. The alternating hydrophobic regions are not glycosylated and do form coils. As a consequence of these alternating hydrophilic and hydrophobic regions, mucin fibres have the ability to catch both, hydrophilic and hydrophobic particles by forming low affinity bonds. Single mucin fibres do also form bonds between each other which leads to a steady rearrangement of the mucin layer [86,91,92]. 

#### 4.2.2. Mucus Permeability and Turnover

Due to its properties, mucus is not only able to adsorb exogenic particles like pathogens. Analogously drugs can be cleared very fast which limits their bioactivity. Effective drug formulations developed for intranasal administration should therefore penetrate the nasal mucus and adhere to the local epithelium to minimize mucocilliary clearance. Permeability of mucus depends on the properties of the invading substance, mucus thickness as well as mucus consistency. Once the mucus is passed, the nasal epithelium provides a rather good permeability [93]. 

It is thought that small particles like protein degradation products and viruses move quite freely in mucus [94]. Mesh space of mucin ranges from 20 to 200 nm and is clearly wide enough for small molecules and particles, while bigger particles are slowed down by the mesh [95]. Mucus mesh space will not hinder smaller drug particles from diffusing through mucus. Uncharged drug particles, which do not interact with mucus are able to diffuse through mucus with a speed similar to their diffusion velocity in water [96]. Hydrophilic drugs will pass mucus more easily than lipophilic drugs. There are electrostatic, lipophilic and Van der Waal’s interactions. Positively charged ions bind to negatively charged mucins, but this interaction is pH dependent and is weaker in ions of high ionic strength [1,96]. 

Interactions between molecules and mucus are crucial, known from the IgM-mucus interaction. IgM is so small it could pass mucus freely, but in cervix mucus it is nevertheless slowed down due to low affinity bonds occurring between mucus and Fcµ domain of IgM antibodies [94,96]. Mucosal interaction with the Fc domain of antibodies results in reduced transport rate and enables thereby the antibodies to trap pathogens which otherwise would diffuse through mucus freely [86]. A similar interaction with Fcγ domains needs to be elucidated as it could interfere with intranasal delivery of monoclonal antibodies. 

Microbiological studies have shown that thicker, more viscous mucus immobilizes bacteria better than less viscoelastic mucus. Leukocytes are able to migrate through normal, less viscous mucus and perform immune defence, but they are not able to penetrate high viscous mucus. Thickened mucus slows mucus clearance. This in turn leads to increased residence time for bacteria to penetrate mucus barrier and simultaneously impairs immune cell immigration and killing performance [86,97,98]. 

An increase of mucoadhesion is thought to increase drug uptake and hence bioavailability by prolonging the residence time in the mucus. Another approach is to increase the adhesion to epithelial cells layer, but it should be noted that drug or particulate formulation with encapsulated drugs have first to pass the mucus to adhere to the epithelium. From studies with intestinal mucus it is known that excipients can be used that disturb the mucosal structure to enable a better diffusion of the drug. Nevertheless, it has to be considered that such manipulation would also affect mucociliary clearance with all associated consequences [1,96]. However, as the nasal mucus layer is considerably thinner than the intestinal mucosal layer, the problem of permeating the mucus layer is less pronounced for intranasal delivery. 

A secretion of 20 to 40 mL of nasal mucus was reported under normal conditions per day [99]. The mucus is propelled by motile cilia of the epithelial cells to the nasopharynx where it is swallowed and subsequently digested [100]. As detailed above, mucus entraps particles and bacteria and forms micelles containing exogenous substances, which can then be removed [86,101]. Hence, the clearance of pathogens is dependent of mucus degradation. Transport velocities of mucus range from 1 to 2 mm/h in the anterior portion of the inferior turbinate and up to 8–10 mm/h in the posterior portion of the inferior turbinate [99]. The thickness of the mucus layer depends on the rates of secretion and its degradation. Even un-propelled mucus is automatically renewed by continuous mucus secretion [86]. 

#### 4.2.3. Physico-Chemical Properties and Mucociliary Clearance

**Figure 6:**
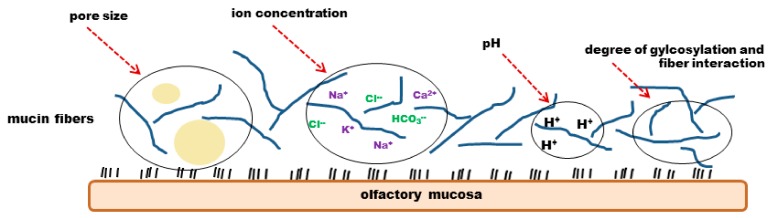
Physicochemical properties of mucus. The viscoelasticity and permeability of mucus is dependent of the degree of glycosylation and fibre interaction, as well as of the pore size, ion concentration and pH.

Mucus can be divided into two different layers, the pericilliary layer adjacent to the epithelial cells and the upper gel like layer. Pericilliary layer is of low viscosity and reaches nearly as high as the tips of motile cilia. In the intestinal mucus, the upper, more viscous layer is between 0.5 and up to 5 µm thick but it can be estimated that the layer is thinner in the nasal mucus, but exact data are still missing. The most rigid layer of mucus is found adjacent to the glycocalyx on the apical side of the epithelium [93]. Mucus displays non-Newtonian properties. This means it possesses both, viscous (fluid) and elastic (solid) properties, termed viscoelastic [93]. If shear stress is applied to mucus, its viscoelasticity ensures decreasing viscosity with increasing shear stress. Recovery of mucus is only partially after removing stress [102]. Viscidity describes the property of mucus to adhere to surfaces and to form low affinity bonds with exogenic particles [86]. Mucus is a dynamic, semipermeable gel as it contains not only glycoproteins, but also inorganic salts, lipids, scraps of DNA, other proteins such as immunoglobulins, enzymes and debris (Figure 6) [86,96,103,104]. Besides, the presence of these molecules, the mucus texture is also dependent on pH and ionic strength. Ions as Na^+^, Cl^−^, K^+^, Ca^2+^ and HCO_3_^−^ contribute to the ion strength [105] and contribute to a viscosity of 1.6 ± 1.5 Pa·s [106]. Several data indicate a mucosal pH from 5.5 up to 7.8 in the nose [82,107,108]. On average nasal mucus is cleared every 10 to 20 min [109]. Cilia are only able to transport mucus with appropriate viscoelasticity. If mucus is too slippery it cannot be propelled by cilia and drips out of the nose or down the lungs, respectively. If it is on the other hand too viscous and sticky, it will not be propelled either [86].

Mucociliary clearance can be influenced by drugs as they may inhibit or increase ciliary beat frequency. In general, anaesthetics are inhibitors of mucociliary clearance. Partly or even complete irreversible ciliostatic effects were shown by cocaine and lidocaine, respectively [93]. In addition, cholinergic antagonists, alpha-adrenergic receptor agonists, corticosteroids and anti-histaminic drugs also inhibit mucociliary clearance, while beta-adrenergic agonists and cholinergic agonists increase the ciliary beat frequency [93].

#### 4.2.4. Aquaporins as a Mucosal Source of Water

As the nasal mucosa is constantly exposed to the inhaled air it is vulnerable to dehydration. AQP are ubiquitous expressed membrane associated water channel proteins. They are involved in transfer of water, small solutes and ions across the cell membrane in a bi-directional manner [46,51]. AQP have a high selectivity and capacity [110]. Up to date, there are 14 different AQP identified [110]. AQP1 is expressed in epithelial cells of blood vessels and surrounding connective tissue cells in both, olfactory and respiratory mucosa. Thus, it may be involved in water transport of blood vessels [51]. AQP1 is also expressed in OEC [111] and on fibroblasts surrounding Bowman’s glands [46,51] while AQP4 is expressed in plasma membranes of supportive cells. Takata et al. examined AQP distribution in the respiratory and olfactory mucosa of rats. AQP1, AQP3, AQP4 and AQP5 were found in the nasal mucosa. While AQP1, AQP4 and AQP5 are specialized for water transport, AQP3 belongs to the subfamily of aquaglyceroporins and is not only permeable to water, but also for small solutes as glycerol and urea [51]. AQP3 and AQP4 were found in the basolateral membrane of Bowman’s glands. In contrast to that, AQP5 was found on secretory acinar cells in the apical membrane of Bowman’s glands. AQP4 is expressed in Bowman’s glands, basal cells as well as epithelial cell of the olfactory mucosa. Verkman et al. showed that mice lacking AQP4 were impaired in olfaction due to altered neuroexcitation indicating an important role of AQP4 in olfaction [50]. Taken together, AQP3, AQP4 and AQP5 may participate in the maintenance of the specific microenvironment needed for odorant reception in olfactory dendrites [51]. 

## 5. Intranasal N2B Drug Delivery of CNS Active Substances

### 5.1. Mechanisms of Intranasal N2B Drug Delivery

As demonstrated above, the nasal mucosa and especially the olfactory mucosa are highly suitable for drug delivery. Drug transport via neuronal connections appears to be the most relevant pathway to reach the CNS. In particular, two possible mechanisms for intranasal drug uptake are discussed: the intracellular and the extracellular pathway [3,10,11]. The intracellular pathway includes endocytosis of the drug into olfactory or trigeminal axons and transport inside the nervous tissue until they reach a synaptic cleft in the olfactory bulb or the brain stem, respectively. The drugs can travel along the nerve in endocytic vesicles. When reaching the brain the drugs are exocytosed and can distribute throughout the CNS [3,10,11]. Via this pathway, both, the olfactory and trigeminal nerves were shown to endocytose viruses and bacteria resulting in CNS infections [11,72]. In particular, the olfactory nerve is able to endocytose particles with a high variety in size. It is capable of internalizing small molecules like aluminium lactate (294 Da) as well as wheat-germ agglutinin horseradish peroxidase (WGA-HRP, 80 kDa). The intake of WGA-HRP is most likely receptor mediated, although receptor mediated endocytosis of molecules is rather the exception than the rule [11]. It was shown that the trigeminal nerve endocytoses intranasally applicated drugs via the ophthalmic and the maxillary branch [10,11]. However, the process of neuronal translocation is very slow: it was reported that the transport via the olfactory nerve takes 1.5–6 h, while the transport via the longer trigeminal nerve was described to take 17–56 h. Since there are several studies confirming a rapid N2B delivery within minutes only, the intracellular pathway is apparently one route, but not the primary route [11,68].

**Figure 7:**
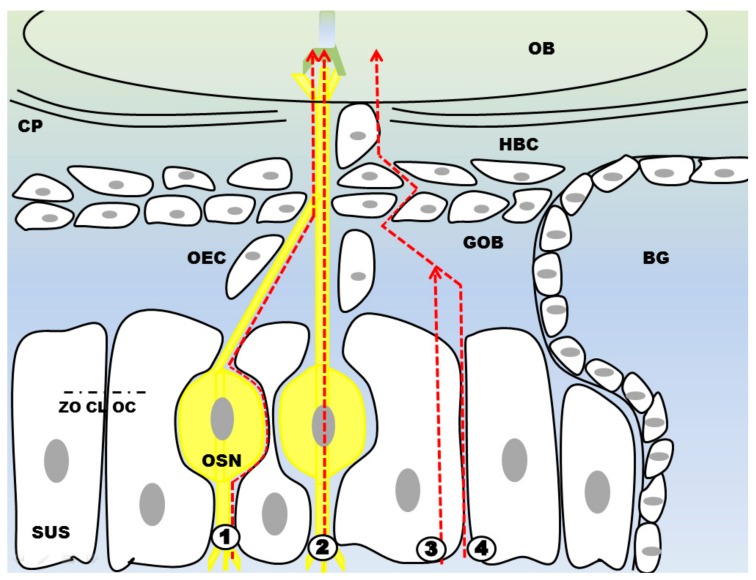
Four different routes have been described for N2B drug delivery. The extracellular pathway ① directs the drug to the CNS along the OSN (or trigeminal nerve which is not shown here) via bulk flow processes. For the intracellular route ② the drug is endocytosed and then shuttled to the CNS where it is finally exocytosed. ③ and ④ describe drug transport through or along supporting cells. The drug molecule can either be endocytosed by supporting cells or travel through the intercellular space. By travelling through the intercellular space ④ the drug has to pass tight junctions like zonula occludens (ZO), claudin (CL) and occludin (OC). It should be noted that intranasal drug delivery is rather a mixture of these different pathways than being limited to one only. Abbreviations: SUS: sustentacular cells; OSN: olfactory sensory neuron; OEC: olfactory ensheathing cell; GOB: globose basal cells; HBC: horizontal basal cells; BG: Bowman’s gland; CP: cribriform plate; OB: olfactory bulb.

The extracellular pathway ① is described along olfactory or trigeminal nerves by bulk flow processes [10,11]. Thus, the drug is crossing the epithelial layer and when reaching the lamina propria the drug is included into the cleft between the axons and the ensheating layer. To reach the nerves, the drug has to pass the epithelial tight junctions, which could limit the uptake. However, this hurdle may be porous, as the neuronal turnover within the olfactory epithelial layer is rather high. As described above, the olfactory neurons have a short lifetime until they undergo cell death and leave a gap [2,3,11,96]. The new neuron regrows in this gap, but these clefts may allow an efficient drug uptake even for larger particles. In addition, the formation of tight junctions lining the apical layer was reported to be delayed [11]. Two other pathways along or across sustentacular cells with apparent less importance are depicted in Figure 7.

It should be noted that the passage through the lamina propria does not consequently imply that the drug will arrive in the CNS. It may also be absorbed by blood vessels and in turn enters the systemic circulation. In addition, it may enter glands or lymphatic vessels connected to the deep cervical lymph nodes. Finally the drug can also enter the cranial nerves via extracellular diffusion [3,10]. Taking the trigeminal or olfactory route, it can reach the subarachnoid space adjacent to the pons or the olfactory bulb. The further distribution of the drug in the CNS appears to be mediated via bulk flow of the CSF [3,10,11,112]. In rodents, the distribution may be mediated via the rostral migratory stream [3,11].

The intranasal transport of drugs to the brain does apparently not follow one specific route only. It is more likely that a substance can be taken-up by several of the above-mentioned pathways in parallel (Figure 7). The translocation within neurons, as describe in route ② is rather slow. Thus, it is most likely not the primary route of delivery, as it has been shown that the N2B delivery is quite fast [11]. The mean velocity of axonal transport was measured 25 mm per day. The olfactory nerve of mice is about 4 mm in length and the trigeminal nerve is about 20 mm in length [113]. It seems that the intracellular transport is not predominantly size-dependent as is it was shown that the transport velocity for small and high molecular weight particles was similar. However, endocytosis (② and ③) is a process dependent on the molecular weight of the substance as long as it is not receptor mediated [11,113]. Regarding the extracellular way of N2B delivery Thorne et al. estimated between 0.73 and 2.3 h for a substance to travel from the olfactory region to the olfactory bulb. The same molecule was estimated to take 17 to 56 h to reach the pons traveling alongside the trigeminal nerve. Comparing travel times of the substance, the olfactory pathway is the more feasible route [3,11]. There are arteries running along the olfactory axon bundles, providing nutrient supply for the olfactory sensory neurons. Drug substances will be most likely moved via the perivascular pump by systolic-associated high pressure wave moving through the arterioles. This leads to a rapid nose-to-brain transport velocity and enables drug substances to reach the central nervous system after 0.33 h, taking the olfactory pathway and 1.7 h taking the trigeminal pathway, respectively [3,11]. 

### 5.2. Limitations of Nose-to-Brain Delivery

Direct N2B delivery of therapeutic substances is reported to potentially have a wide range of advantages, such as bypassing the blood-brain barrier or the increased patient’s compliance compared to intrathecal delivery. However, N2B targeting does also have its limitations: exacting dosing of intranasally applied drugs is still an unsolved challenge, which is one of the reasons why intranasal insulin (Nasulin^®^) did not replace subcutaneous insulin injections [12]. Intranasally applied substances undergo a rapid elimination by mucociliary clearance and by drainage to the lower part or to the pharynx. This may vary with the dosage form. Furthermore, the anatomy of individual nasal cavities is quite different. Therefore, administration techniques display a broad variation and need to be tailored to the individual anatomical characteristics.

Moreover, the dosage volume is restricted as the upper parts of the nasal cavity are rather small and narrow [114]. Regarding the formulation, it is important not to use substances with mucosal toxicity or substances causing irritation or allergic reactions. The health status of the patient is also important, as there may occur some problems with the functionality of intranasal delivery devices in patients suffering from allergies or who have a cold. Furthermore, it should be ensured that the frequent use of these devices does not harm the nasal mucosal surface. Another great impact for safe and efficient N2B targeting is the reproducibility of drug administration in the olfactory region [115]. The adjacent regions, as the respiratory region, are highly circulated with blood and thus favour a systemic uptake. Another unsolved issue is the delivery of high molecular weight substances. Although, some studies report a rather weight-independent transport, the permeation of the epithelial barrier of endocytosis usually depend on the hydrodynamic radius. Substances smaller than 400 Dalton will diffuse freely while substances of 1000 Daltons or more appear to stuck in the mucus. These substances have to be linked to a ligand enhancing bioavailability. Once the CNS is reached, there is still the problem of targeting the drug to the specific site of action [114,115,116]. Nevertheless, N2B delivery is a promising alternative for CNS drug delivery and more high-quality research can find suitable solutions for the above-motioned limitations.

## 6. Formulations, Dosage Forms and Medical Devices for Intranasal Delivery

N2B delivery provides a great opportunity for fast and patient compliant drug application. As demonstrated above, there are good reasons to assume that targeting the olfactory mucosa would increase the chances to deliver drugs to the CNS. Although, regarding the application of the drug, there are still some limitations to overcome as the olfactory region and in particular the olfactory cleft is well hidden deeply in the nasal cavity. Furthermore, since the olfactory cleft is at the roof of the nasal cavity, formulations need a rather good adhesion to stay on the mucosa.

### 6.1. Excipients

#### 6.1.1. Mucoadhesive Excipients

Mucoadhesive polymers like chitosan, hypromellose, carbopol, carboxymethylcellulose, polyacrylic acid or others have been extensively used as excipients for intranasal formulations (for summary see [117]). Most mucoadhesive molecules are positively charged to build-up interactions with the negatively charged mucins. As reviewed above, most mucins are bound to the apical surface of epithelial cells. By adhering to such cell bound mucins, these agents prolong the residence at the mucosa and, thus, improve drug uptake. Although, throughout the literature mucoadhesion is synonymously used for increased residence time, it can be well suspected, that mucoadhesive agents that bind to the secreted mucus are cleared rather quickly. Nevertheless, when targeting the olfactory mucosa mucociliary clearance does presumably not play a major role as motile cilia are absent. But mucociliary clearance should not be neglected as clearance still occurs by secretion from Bowman’s glands, gravitational forces and due to the presence of islets made from respiratory mucosa [46,79,118].

Chitosan, a copolymer of *N*-acetyl-d-glucosamine and glucosamine, is already used as supplement to weight loss products, as it attaches to fat in the stomach and prevents its absorption. Characteristics of chitosan are its biocompatibility, low toxicity and biodegradability. Chitosan is interesting for drug delivery as it has also mucoadhesive properties and enhances the penetration of the cellular membrane [119]. There are a lot of chitosan derivatives as N-trimethyl chitosan with enhanced solubility over broader pH ranges, or carboxymethylated chitosan, which is able to form viscoelastic gels [119].

#### 6.1.2. Adsorption Enhancers

Excipients that improve permeation and absorption are e.g., cyclodextrins, bile salts, laureth-9 sulfate, fusidate derivates, fatty acids, hydrophilic polymers, surfactants etc. [120]. In particular, methylated β-cyclodextrins are used to enhance the absorption of poorly water soluble, lipophilic drugs since they form inclusion complexes with the drug. Absorption enhancers can have a reversible effect on ciliary beat frequency [93].

To reach the olfactory mucosa and thus the CNS, intranasally applied drugs have to penetrate the mucus. Negatively charged and hydrophilic excipients do not interact with mucus, whereas positively charged and hydrophobic agents display mucus interaction. To design drug formulations which penetrate mucus easily, poly(lactid-*co*-glycolid) acid (PLGA) can be used for nanoparticles. Further, PEGylation eliminates hydrophobic interactions of the particle with mucus. For particles, it is important, to ensure hydrophilic characteristics by a regularly coating [121].

#### 6.1.3. Preservatives

To prolong stability of nasal drug formulations preservatives are used. These preservatives do also affect mucociliary clearance as most of them display ciliostatic or ciliotoxic effects. Reversible cilioinhibition was shown by lipohilic preservatives such as chlorobutol and hydroxybenzoates as well as methylhydroxybenzoates, propylhxdroxybenzoates and chlorobutol used in aqueous drug formulations. Whereas chlorocresol, edetate, benzalkonium chloride, phenylmercuric acetate and thiomersal caused irreversible damage. Phenylmercuric acetate and thiomersal were shown to be more ciliostatic than benzalkonium chloride [93,122,123]. However, a study published by Rijntjes et al. focusing on the long-term administration of 0.02% benzalkonium chloride as preservative proved the safety of the drug supplement as it did not cause changes in nasal mucosal morphology or effects on mucociliary clearance [93,124]. Menthol and eucalyptol which are used in some decongestants displayed strong ciliostatic effects [93,125]. It was also shown that preservatives used in decongestants as xylomethazoline and oxymethazoline do have additive cilioinhibitory effects and may lead to *rhinitis medicamentosa* after long-term abuse [93,126,127].

### 6.2. Formulations and Dosage Forms

#### 6.2.1. Semisolid Formulations

Hydrogels consisting of e.g., chitosan, carbopol, hydroxypropyl methylcellulose, polyvinyl alcohol have been extensively studied for intranasal delivery [128]. Marketed products for self-medication into the lower parts of the nasal cavity contain hyaluronic acid or hypromellose. Interestingly, although not all these agents are mucoadhesive, their suitability to improve bioavailability of intranasally delivered drug had been comprehensively demonstrated [129,130]. In addition, due to their higher viscosity, semisolid formulations have a clear advantage over liquid formulation when targeting the olfactory cleft with its mucosa facing either upside-down or upright. N2B delivery of pharmaceutics is also interesting for neurological diseases like epilepsy, autism spectrum disorder or migraine [14,131,132]. The group of Vyas et al. developed a clonazepam microemulsion and performed a study in rats demonstrating, that the blood/brain ratio of clonazepam was at any sampling point two-fold higher in the nasally administered group than in the group of intravenous injected rats. This may indicate that N2B delivery could be a fast and efficient way to treat acute status epileptics [131]. 

#### 6.2.2. Particulate Formulations

The use of nanoparticles as drug delivery systems allows controlled and site-specific delivery of therapeutic agents. Nanoparticles are able to protect the drug from biological or chemical degradation and help to evade drug-efflux mechanisms as P-gycoprotein transporter in the blood-brain barrier, due to encapsulation of the drug [133]. To ensure the release of the content on the site of action, nanoparticles may be linked to specific targeting ligands [15,133].

Pardeshi et al. described some useful properties nanoparticles should show for efficient N2B delivery: non-toxic, biocompatible, as well as biodegradable, physical stability, cost effective manufacturing process and scale-up, compatible to link with small molecules, proteins, peptides or nucleic acids. Furthermore, formulating into nanoparticles can improve the drug’s stability and reduce excipient-induced drug alterations to a minimum. To reduce the frequency of drug administration and improve patient’s compliance particulate formulations can provide controlled drug release profiles [133]. 

There are three options of tailoring nanoparticle uptake that are suitable for N2B transport. The first one is to enhance the mucoadhesion to prolong the time of nanoparticle interaction with the mucosa. This will enhance the probability of the drug passing the epithelium and, finally, reaching the brain. The second one is the ability of different materials used for particulate formulations to decrease the barrier function of tight junctions. These specialized particles would be able to transiently open tight junctions and thus allow the drug to enter the nasal mucosa. The last opportunity is to enhance the chance of endocytosis of nanoparticles. Once endocytosed, the nanoparticles could release their drug content, which would be further delivered to the brain [68,133,134,135].

The use of nano- and microparticles for drug delivery brings along some benefits, such as a controlled and sustained drug release or by shielding the drug against environmental influences. This is in particular important when delivering proteins and peptides. Remarkably, particles can include ligands or molecular imprinting on their surface which guide them to a targeted disease site [136,137]. In summary, particles may thereby minimize the drug dose required, provide a lower toxicity and decreased adverse effects [1,68]. The challenges are that nanoparticles should on one hand adhere to mucus in order to improve drug absorption but on the other hand should also be able to penetrate mucus to avoid entrapping and clearance of the drug.

There are various particulate drug delivery systems either on the market or in the pipelines [1]. Different nanocarriers can be used, for example polymeric, lipid and inorganic nanoparticles [138]. Agents as chitosan, hydroxypropylcellulose, carboxymethylcellulose, and carbopol display mucoadhesive properties, but also hyaluronic acid and polyacrylic acid are well suited [137]. Hassan et al. showed that thiolated chitosan particles doubled the brain delivery of the anxiety disorder treatment drug buspirone hydrochloride [138]. As chitosan is a highly mucophilic agent, high concentrations can even lead to mucus gel break down what in turn leads to an easy access for the drug to epithelial cells. But it has to be considered that mucus gel break down also impacts pathogen defence [86,138].

Biodegradable drug carriers as poly lactic acid (PLA) or poly glycolic acid and their polymer PLGA have demonstrated a huge potential for intranasal delivery (see [117] for summary). The size of PLGA can be adjusted. Small drugs, peptides, proteins and plasmid DNA can be transported with the help of PLGA nanoparticles. Even the drug release can be controlled by adjusting the molecular weight of PLA, which in turn determines the degradation rate [1]. An additional mechanism for controlled drug release are polyanhydride nanoparticles or to conjugate the drug to a polymeric carrier via a linker [1]. Drawbacks in the use of polymeric nanoparticles may occur during the production process, as contaminations from the production process may occur. These contaminations may result from the use of organic solvents, polymer aggregates, toxic monomers and polymerization initiation substances. Furthermore, the production and sterilization processes are expensive and there are only few techniques available for a large-scale production of nanoparticles [139,140,141,142]. Regarding bioavailability polymeric nanoparticles do have limited capabilities of crossing the blood-brain barrier [68].

Bromocriptine, a promising drug for Parkinson’s disease, acts as an antioxidant and reduces apoptosis. Since its low bioavailability after oral drug application limits its efficacy, intranasal administration of bromocriptine would allow to bypass the first pass effect and to direct the drug specifically to the site of action [4,133]. The group of Shadab et al. loaded chitosan nanoparticles with bromocriptine and were able to show a significant beneficial effect in a mouse model of Parkinson’s disease treated with intranasally administered bromocriptine nanoparticles [4,133,143]. 

N2B can also be used to deliver other substances such as siRNA or DNA to the brain. The specifically targeted delivery of siRNA or DNA to the CNS will open new possibilities for gene therapy of neurological diseases [144].The group of Sanchez-Ramos et al. manufactured chitosan nanoparticles carrying siRNA, which were intranasally administered to rats. Using a reporter gene, they could demonstrate the successful targeting of siRNA to the CNS in several brain regions 24 and 48 h after intranasal administration [144]. 

#### 6.2.3. Lipid-Based Formulations and Liposomes

Lipid-based carriers such as mono-, di-, triglycerides, fatty acids and waxes reduce the toxicity of the drug and may be used for sustained release of the drug. Lipid nanoparticles are highly stable and are able to encapsulate hydrophilic as well as hydrophobic drugs. Lipid-based drug shuttle systems are able to cross the blood-brain barrier easily [68]. Nanostructured lipid carriers are composed of blends of solid and liquid state lipids. Due to their unique properties they have high drug load capacities and maintain drug stability [145]. Liposomes or lipid-based carriers as well as polyethyleneglycol (PEG) are approved and established in clinical practice [1].

#### 6.2.4. Liquid Formulations and Formulations for Intranasal Vaccination

Liquid-based formulation for drugs with local interactions in the mucosa of the lower parts of the nasal cavity e.g., decongestants or sea water/saline are clinically well established. However, such formulations are rather quickly cleared and drain by gravitational forces towards the pharynx, where they can cause undesired adverse effects like a dry throat. Whenever exact doses are needed, liquid formulations are less suited for intranasal approaches as dosing is hindered by mucociliary clearance and gravitation.

Vaccination through the mucosal immune system can be an effective way to stimulate the immune system without using parenteral injections. Vaccination through the nasal route is of special interest in paediatrics since it was successfully shown that nasally administered vaccination is able to induce a systemic immune response [67]. M cells are transcytosing the vaccine antigens very fast and transfer them to the underlying lymphatic tissue. The immune system is then primed and protection against the pathogen is induced [60,64]. However, other ways of immune interaction cannot be excluded. An easy way of intranasal vaccinations is dispersing an ordinary commercially available vaccine for intramuscular administration into the nasal cavity. Although, this is not a very efficient approach, the liquid-based formulation drains and is propelled to the nasopharynx, thereby interacting with the NALT’s M cells. When developing intranasal vaccine formulations, some aspects should be considered to facilitate M cell uptake. Lipids or lipid-based particles can be used as supporting material. Lipids are biocompatible and flexible in terms of formulation. The particle size is also of great interest. Smaller particles (up to 200 nm) appear to be sensed as viruses and taken up by receptor-mediated endocytosis, leading to cellular immune response. Whereas larger particles (more than 500 nm) will be taken up via phagocytosis or pinocytosis and will most likely induce humoral responses. Regarding nasal administration, smaller particle will more likely reach the nasal mucosa while larger particle will be incorporated by respiratory macrophages [146]. Targeting specific M cell receptors may accelerate the vaccine uptake [60]. A promising route of vaccine administration is to couple the vaccine to IgA in order to induce an immune response. With the aid of IgA there is apparently no need for an additional adjuvant [67]. 

It should be noted that excipients affecting mucociliary clearance may also have an effect on NALT. If mucociliary clearance is impaired toxic substances or pathogens may reach the lymphatic tissue to a greater extent than normal and provoke immune reactions and on the other hand may an increased mucociliary clearance prevent substances to reach NALT [147].

### 6.3. Intranasal Medical Drug Delivery Devices

Nasal pump sprays and droppers are most likely the best-known nasal medical devices, but they are limited to liquid-or lipid-based formulations. But even if different application methods, as changing of the head position were used, the intranasally applied liquids do hardly reach the olfactory cleft. If the drug is not able to reach the olfactory region efficiently, it has a reduced chance to be transported to the CNS, but cleared by mucociliary clearance or absorbed systemically by blood vessels instead [148,149,150]. 

Inhalation with nebulized liquid- or powder-based aerosols fails to target the olfactory cleft efficiently [151]. Therefore, some devices were developed to enable N2B drug delivery. Devices as the electronic atomizer ViaNase^TM^ or the bidirectional breath-powered nasal delivery device called OptiMist^TM^ that both, deliver drugs to a larger surface of the nasal cavity than pump sprays and droppers do. The OptiMist™ device consists of a mouthpiece and a conical, sealing nosepiece. It contains of a pump-spray and an additional breath-activated mechanism to create 43-µm droplets. To apply the drug, the OptiMist™ device is inserted into one of the nostrils and the patient blows into the mouthpiece. By blowing into the mouthpiece the soft palate is closed and the oral pressure is transferred to the nostril. To balance the pressure, the air exits through the other nostril, which is then called a bidirectional flow. During the patient exhales, particles are released. As the particles are released at exhalation even small particles will not enter the patient’s lungs. OptiMist^TM^ is able to deliver the therapeutic agent into the upper posterior sector of the nose and at the same time reduces drug deposition in the lower nasal segments [152,153,154,155,156]. 

The ViaNase™ device is a vortex-propelled nebulizer system. ViaNase™ consists of a sealed nosepiece and a device where an active vortex of nebulized particles is created. ViaNase™ device is able to generate droplets with a diameter of 9–11 µm [153].

The so-called Precision Olfactory Delivery (POD^®^) device aims to deliver drugs specifically into the upper nasal cavity operated by pressure. Manufacturer’s data claim a 50% deposition in the olfactory region [157], but independent studies are still missing. 

While the above-named devices are suitable for particulate formulations, there are hardly any devices available for the efficient administration of viscous semisolid formulations like hydrogels.

## 7. Conclusions

N2B delivery of therapeutic agents is a future perspective to treat neurological diseases. To successfully create N2B delivery systems one has to understand the unique structure of the olfactory region. 

The use of N2B delivery mechanism in drug application has some advantages over strategies that aim to cross the blood-brain barrier. It can be assumed that with the use of N2B a lower dosage of the applied drug is needed, as the transport is directed specifically from the nose to the brain and has not to overcome blood-brain barrier obstacles and systemic clearance. The specific targeting leads also to a reduced risk of systemic toxicity. A major point of N2B is the rather high patient compliance. 

To develop suitable intranasal formulations, but also medical delivery devices, some hurdles have to be cleared. Especially, avoiding immunogenicity of biopharmaceutical proteins is a challenge that can be only solved by an intelligent formulation strategy. Up to now, the olfactory mucosa and in particular the olfactory mucus have both been inadequately characterized in the literature. Medical devices that target specifically this site are still missing. Thus, further research is needed to advance intranasal N2B delivery and to path its way to save and reliable clinical application.

## Figures and Tables

**Table 1:** Overview of drug delivery pathways related to the nasal cavity.

Drug Delivery Route Related to Different Nasal Mucosa		Examples with Supporting Clinical Data
local administration	predominantly squamous and RE	decongestants, local anaesthetics, glucocorticoide [19,20]
systemic delivery	predominantly RE	calcitonin, sumatriptan, desmopressin [21,22,23]
intranasal vaccination	NALT and immune cells in all mucosal types	seasonal flu vaccine [24,25]
CNS delivery (N2B)	OE: olfactory neuronal bundles; RE/OE: trigeminal nerve endings	oxytocin, insulin [13,14]

RE: respiratory mucosa; NALT: nasopharynx-associated lymphatic tissue; CNS: central nervous system; N2B: nose-to-brain; OE: olfactory mucosa.

## Abbreviations

AQP	aquaporin
BG	Bowman’s gland
CNS	central nervous system
CSF	cerebrospinal fluid
GOB	globose basal cell
HBC	horizontal basal cell
HIV	human immunodeficiency virus
Ig	immunoglobulin
MALT	mucus-associated lymphatic tissue
M cell	microfold cell
MUC	mucin
N2B	nose-to-brain
NALT	nasopharynx-associated lymphatic tissue
OC	occludin
OE	olfactory epithelium/olfactory mucosa
OEC	olfactory ensheathing cell
OSN	olfactory sensory neuron
Pa·s	Pascal·second (unit of viscosity)
PEG	polyethylenglycol
PLA	poly lactic acid
PLGA	poly(lactid-*co*-glycolid) acid
RE	respiratory epithelium/respiratory mucosa
SUS	sustentacular cell
ZO	*zonula occludens*
